# Navy Medical Board

**Published:** 1859-07

**Authors:** 


*Navy Medical Board.*—The Board of Naval Medical Examiners, consisting of Surgeons W. S.W. Ruschenberger, L. B. Hunter, J. D. Miller, and Passed Assistant Surgeon George H. Howell, adjourned *sine die*, May 23d, 1859.

Assistant Surgeons Thos. J. Turner (appointed in 1853), R. P. Daniel, William G. Hay, and William T. Hord (appointed in 1854), were found qualified for promotion.

Of the competitors before the Board for the ten vacancies which will probably occur, in the course of the year, in the grade of assistant surgeon, the following have been selected :—

1, William Bradley, Pa.; 2, Edward F. Corson, Pa.; 3, David Kendleberger, Ohio; 4, Joseph D. Grafton, Arkansas; 5, Robert L.Weber, Pa.; 6, Robert J. Freeman, Va.; 7, William E. Taylor, Va.; 9, James McMaster, Pa.; 10, James W. Herty, Ga.

The five first named have been already appointed, and it is probable the others will receive commissions in the Medical Corps of the navy prior to the meeting of another board.

It is supposed that the increased activity of the naval service, in connection with the comparatively small number of medical officers, a large proportion of whom, from age and physical disability, are incapable of active duty, will induce the next congress to authorize a considerable augmentation of the medical corps. Should this conjecture prove to be correct, it is probable that from forty to fifty assistant surgeons will be required to fill new appointments, in the course of the year 1860. Those young members of the profession, who are desirous to obtain admission into the Naval Medical Corps, should begin now to prepare for the competition which will be open in the course of the next spring.

